# Exploring coronavirus cell entry with functional viromics

**DOI:** 10.1128/jvi.01728-25

**Published:** 2026-04-06

**Authors:** Victoria Jefferson, Ariel Endlich-Frazier, Michael Letko

**Affiliations:** 1 Paul G. Allen School for Global Health, Washington State University 663014, Pullman, Washington, USA; Universiteit Gent, Merelbeke, Belgium

**Keywords:** coronavirus, zoonosis, tranmission, spill over, viral entry, receptor, bat, hedgehog, pangolin

## Abstract

Over the past 2 decades, viral discovery has uncovered thousands of novel coronaviruses in wildlife, including viruses with high similarity to known human pathogens. As human coronaviruses emerge from cross-species transmission events involving wildlife and humans, their frequent discovery in wildlife suggests these viruses will continue to impact global health. Unfortunately, while viruses are discovered often, laboratory limitations have stymied research on experimentally assessing their zoonotic potential. Thus, new laboratory approaches and research mindsets are needed to functionally characterize the ever-growing virome. Here, we discuss several platforms we have developed and the resulting advancements made toward functionally annotating the virome, which we refer to collectively as “functional viromics.” We also explore how these approaches may be adapted to assess other viral phenotypes and how laboratory-derived data sets on viral functions will improve next-generation models of zoonotic risk.

## INTRODUCTION

In November of 2002, patients presenting atypical pneumonia were admitted to hospitals in Guangdong Province in the People's Republic of China (1). By April of 2003, a novel causative agent was isolated from patient samples and named severe acute respiratory syndrome coronavirus (SARS-CoV) (2). Within 8 months, SARS-CoV transmitted via fomites and respiratory droplets across Asia, Europe, and North America, leading to 8,096 cases and 774 deaths (9.5% mortality rate). Conventional public health measures, including screening and patient isolation, lead to the rapid containment of the virus by July 2003 (3). While the exact origins of the virus remain unclear, SARS-CoV was isolated from Himalayan palm civets and a raccoon dog at an animal market in Guandong (4), and epidemiological studies have implicated transmission between humans and animals (5). In 2005, closely related viruses were identified in Chinese horseshoe bats (6), providing additional evidence for zoonotic origins of the virus that emerged in humans just a few years prior. Since these early reports, virus discovery and ecology efforts have gained momentum in the research community, leading to the identification of hundreds of SARS-related viruses in bats, including viruses that enter human cells through the same route used by SARS-CoV-1, which was originally detected in humans (7–9). The emergence of Middle East respiratory syndrome coronavirus (MERS-CoV) in humans approximately 10 years later (10), with an even higher mortality rate than SARS-CoV and origins stemming from dromedary camels, has further driven interest in understanding the animal origins of human coronaviruses (11–14). Coronaviruses have continued to spill over from animals to humans, with a variant of canine coronavirus (CCoV-HuPn-2018) detected in a child with pneumonia in Malaysia in 2018 (15), the SARS-CoV-2 pandemic in 2020 (16), and the 2021 report of porcine deltacoronavirus infection in children in Haiti (17). Other notable coronavirus emergence events include bat to pig transmission of swine acute diarrhea syndrome coronavirus (SADS-CoV) in 2017 (18) and a recombinant feline coronavirus with high pathogenicity in cats in 2023 (19, 20). Given the high prevalence and diversity of coronaviruses and their host species, cross-species transmission events will continue, and coronaviruses will persist as a global health challenge.

Advances in next-generation sequencing technologies, in tandem with a growing focus on uncovering zoonotic viruses, have led to a surge in viral discoveries since the initial SARS-CoV outbreak. While viruses of all types have been reported in wildlife, predominantly in bats and rodents, many belong to the coronaviridae family (21). Unfortunately, most virus discovery studies are performed with viral sequencing as the end goal. As a result, the small sample volumes collected from wildlife preclude experimental efforts to isolate viruses, preventing in-depth characterization of the newly discovered viruses. A lack of knowledge regarding viral culturing conditions represents an additional knowledge gap further hindering viral characterization. Thus, while researchers are aware of viruses circulating in nature, basic questions regarding their threat to humans remain largely unexplored in the laboratory.

## CORE CONCEPT OF FUNCTIONAL VIROMICS

Historically, the study of viruses that have not been isolated has required synthetic biology: producing all or part of the viral genome in question with synthesized DNA to be used in downstream assays of viral functions. Given the large number of uncharacterized viral sequences, it is not feasible to synthesize whole genomes of hundreds, or possibly thousands, of viral discoveries. Because most uncharacterized viruses are presumed to pose little zoonotic or global health risk, the resource burden required to test all discovered viruses for their risk to humans is intractable. Therefore, decision pipelines and methodological approaches are needed to identify and subsequently test portions of uncharacterized viral genomes that are associated with zoonotic risk. In other words, what is the *smallest* region of a coronavirus genome that can be studied under experimental conditions to begin an assessment of the overall zoonotic risk posed by that virus? This question underlies the concept of “functional viromics,” which we define as large-scale, comparative laboratory assessments of viral function for uncharacterized viruses. Ultimately, the goal of this work is to describe viral phenotypes across the global virome.

A primary species barrier for coronaviruses is at the level of cell entry, which is an early viral lifecycle step required for infection. This species barrier is experimentally apparent in the development of animal models. Mice and rats, which are otherwise refractory to infection with coronaviruses from non-cognate species (primarily human and porcine), can be rendered susceptible to viral infection and disease when provided with the appropriate receptor, either transgenically (22–35) or transiently (36–39). Post-entry barriers in these model species, should they exist, are not significant enough to block infection once the viral receptor is present. While entry is certainly not the only barrier faced by coronaviruses, a focus on coronavirus entry can provide key insights regarding their cross-species infection potential.

Coronavirus entry is a multi-step process involving viral attachment to cells, receptor engagement, spike processing by host-cell proteases to free the membrane fusion machinery, and membrane fusion; each step dictated by a unique domain in the viral spike glycoprotein (40–43). Previous studies have demonstrated that coronavirus entry can fail if the spike protein is incompatible with host cell proteins involved in these individual steps (44–47). For example, the N-terminal region upstream of the receptor binding domain (RBD) mediates lectin-directed cell attachment for MERS-CoV, and a mutation in this region results in a cell entry failure (44). Rp3, a bat coronavirus that is similar to SARS-CoV, cannot interact with human ACE2 but can infect cells when provided with just the RBD from SARS-CoV (45). The bat coronavirus HKU4 can use the same receptor as MERS-CoV, but its spike protein is not processed by cellular proteases in human cells as efficiently, resulting in a virus that requires exogenous trypsin to mediate entry (46, 47). Of all the steps outlined above, the interaction between the RBD and host receptors is the best studied interaction for coronaviruses, and assays are well established to measure viral entry in a variety of different cell line backgrounds. The RBD:receptor interaction serves as an ideal starting point for assessing the potential for virus species spillover to new species. Therefore, our initial functional viromics approach has been centered around this tangible and well-defined virus-host interaction.

**Fig 1:**
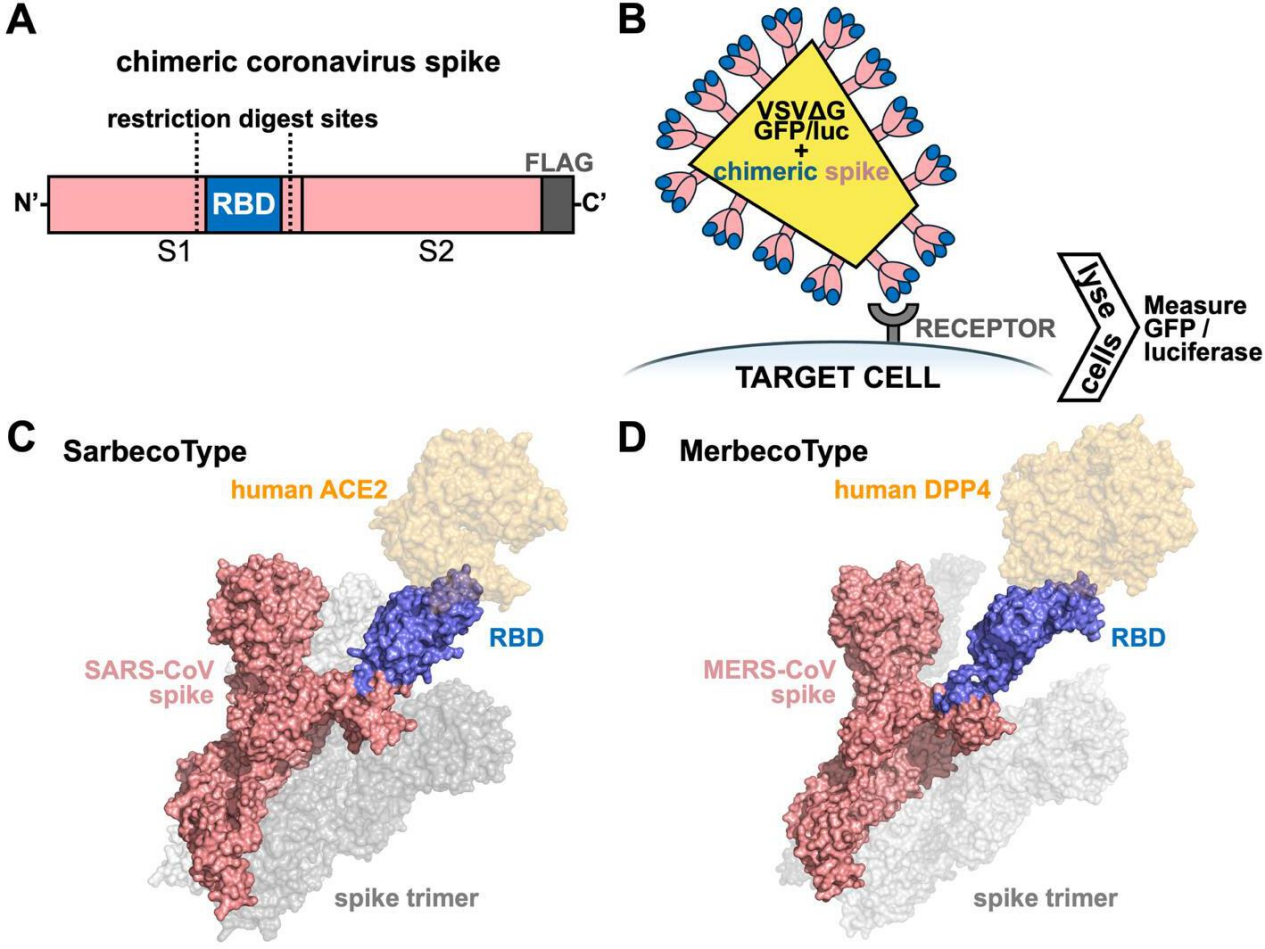
Overview of a functional viromics approach to testing coronavirus cell entry phenotypes. (**A**) Coronavirus spike gene appended with a C-terminal FLAG tag for protein detection and modified with silent mutations that introduce restriction digest sites flanking the RBD. Removal of the original RBD sequence allows for replacement with synthetically produced DNA fragments encoding the RBD from other related viruses, resulting in a chimeric spike. (**B**) VSV with reporter genes, such as GFP and luciferase, is pseudotyped with the chimeric spikes and used to infect target cells from species of interest or expressing defined viral receptor proteins. (**C**) Co-structure of SARS-CoV spike monomer (in light red) bound to human ACE2 (in orange; PDB ID: 5X5B; 2AJF). (**D**) Co-structure of MERS-CoV spike monomer (in light red) bound to human DPP4 (in orange; PDB ID: 4L72, 5X59). In both panels **C** and **D**, the spike RBD is colored in blue, and the other two monomers comprising the spike trimer are shaded in gray.

To test the cell entry functions of coronavirus RBDs, we developed a “generic” spike with a swappable RBD region. At the level of recombinant DNA, we introduced mutations into the coding sequences flanking the RBDs of either the SARS-CoV or MERS-CoV spike. These mutations do not alter the amino acid coding sequence of the spike gene but rather introduce motifs for restriction enzymes in the DNA, allowing for the RBD of the recipient spike to be removed and replaced with synthetically produced DNA encoding for the RBD from a donor spike sequence (Fig. 1A). The RBDs for sarbecoviruses are approximately 500–600 base pairs in length, while the RBDs for merbecoviruses are approximately 700 base pairs. Commercial synthesis of these fragments is more cost and time efficient than a full-length viral genome or even a full-length spike gene. Sequence complexities arising from GC-rich regions and other secondary structural elements often found in the C-terminal region of coronavirus spikes and elsewhere in the coronavirus genome can complicate gene synthesis, requiring mutations or splitting the region into various fragments for downstream assembly. By simplifying DNA synthesis to just a small region of the spike gene encoding the RBD, multiple viruses can be tested in parallel for cell entry phenotypes in very little time and within a reasonable cost. From a biosafety perspective, the use of codon-optimized viral sequences significantly reduces any risk of recombining with natural viral sequences at the nucleotide level. Our SarbecoType platform for assessing sarbecovirus RBD function proved valuable in speed, allowing us to experimentally demonstrate ACE2 receptor use for SARS-CoV-2 just 12 days after the viral genome was published (48, 49). Notably, other methods to test coronavirus standalone RBD function involve more biochemical approaches using purified protein fragments or yeast-two hybrid displays—methods that require knowledge of target receptor identity (50–52). In contrast, the use of chimeric spikes and viral pseudotypes allows for cell entry assessment without prior knowledge of entry pathways and in virtually any cell type that can be cultured under experimental conditions, broadening our ability to assess viral protein function (Fig. 1B). Work with full-length, wild-type spike sequences, while more representative of natural spike proteins, can be complicated by species mismatches with other spike functions, such as host protease processing, which can diminish the ability to detect virus-host compatibility. Some cell lines are also deficient in one or more entry factor, often including host-cell proteases, further complicating assessment of wild-type spike function. To overcome some of these obstacles, many full-length coronavirus spike studies require introduction of either cell-bound or extra-cellular proteases to detect viral phenotypes (53–59). Converse to these approaches, the chimeric spikes we use are derived from human viruses with established cell-culture conditions, which helps retain spike compatibility with cell entry machinery in the common lab cell lines we use in many of our assays. Panels of spike proteins that are identical outside of their RBD regions allow for a focused assessment of RBD function. Using these tools and approaches, our lab has characterized a wide swath of viral discoveries, encompassing the majority of all sarbecoviruses and merbecoviruses discovered to date, providing insights into receptor use and species tropism breadth (48, 60–62).

## SARBECOVIRUSES

Following the onset of the SARS-CoV-2 pandemic, there are now over 9 million sarbecovirus (previously known as betacoronavirus lineage B) entries listed in the National Center for Biotechnology Information (NCBI) database; excluding those that were detected in humans, there are almost 300 SARS-like viral sequences isolated from bats, and more recently, pangolins (63–65). Early work to assess the zoonotic risk posed by bat sarbecoviruses faced challenges in that the viruses did not grow in cell culture or animals (6, 45). A breakthrough occurred when it was discovered that swapping the RBD between a bat sarbecovirus and SARS-CoV could alter virus entry, and this swap alone allowed for the first successful rescue of an infectious, recombinant sarbecovirus (45). Expanding on this early work, we developed a novel functional genomics “plug-and-play” sarbecovirus platform (48). Here, we developed a modular SARS-CoV spike protein with silent mutations to generate easy cloning sites flanking the RBD, permitting the rapid exchange of this minimal functional element (Fig. 1C) (48). We then tested a panel of 30 viral pseudotypes expressing chimeric spikes that represented the total diversity (at the time) of sarbecovirus RBDs for cell entry and receptor usage. Phylogenetic analysis revealed the sarbecovirus RBDs sorted into various clades based on various indels, while our functional assays demonstrated each clade possessed a unique cell entry phenotype (48). Our initial screen confirmed the use of human ACE2 for the known clade 1 RBDs, but not clade 2 or 3 (48). When the protease trypsin was added during pseudovirus infection, a subset of clade 2 RBDs was able to mediate cell entry. (48). These data led us to conclude that this subset of genetically similar clade 2 RBDs was blocked during entry not at the receptor binding step, but at the level of protease processing (48).

During canonical coronavirus entry, the spike protein is cleaved at the cell surface by proteases after the RBD has engaged with the receptor. This proteolytic cleavage allows for conformational changes in the C-terminal domain of the spike, leading to exposure of the fusion machinery and subsequent membrane fusion (66). Spike activation by transmembrane serine protease 2 (TMPRSS2) has been clearly demonstrated for betacoronaviruses, including sarbecoviruses, SARS-CoV and SARS-CoV-2, merbecoviruses, MERS-CoV and bat HKU4, as well as the human ACE2-using alphacoronavirus, NL63 (HCoV-NL63) (47, 67–71). Several of these coronaviruses have been shown to enter cells in the absence of TMPRSS2 through proteolytic activation of the spike in the endosome by cathepsin L (72–74). This alternative, endosomal entry has been directly shown for human betacoronaviruses SARS-CoV, MERS-CoV, SARS-CoV-2, as well as the APN receptor user, human coronavirus 229E (HCoV-229E) (70, 72, 74–76). As many cell lines are deficient in various surface-bound proteases, adding trypsin during pseudovirus infection allows for entry without a host protease. Importantly, even in the presence of trypsin, spike proteins still need to engage with the receptor to infect target cells (48, 57, 58).

In follow-up studies, we have confirmed ACE2-independent, protease-dependent sarbecovirus entry with infectious virus (58). Using our previous findings, we demonstrated that a recombinant sarbecovirus, WIV1, with the spike from clade 2 RBD virus, Rs4081, could be rescued in the presence of high levels of trypsin, independent of ACE2 expression (58). We then applied these findings to develop a simple trypsin-mediated sarbecovirus rescue protocol and were able to isolate and recover RsHuB2019A, a clade 2 sarbecovirus, from *Rhinolopus sinicus* field samples collected in China (59). Additional studies with the spike from ACE2-independent bat sarbecovirus Rs4081 demonstrated that other secreted proteases could promote virus entry, including bacterial thermolysin and elastase, a protease that plays a role in several human lung diseases, as well as the respiratory-tract-specific TMPRSS11A, TMPRSS11D, and TMPRSS1E (56). Taken together, protease processing is a unique challenge for coronavirus assessment but can be accounted for with a variety of approaches.

More recently, we screened an updated sarbecovirus RBD panel, which primarily included additional clade three members from Africa and Europe (60). Here, we found that the sarbecovirus clade 3 RBD from Khosta 2, found in bats in Russia, could robustly mediate human ACE2 receptor-dependent pseudovirus entry (60). When we modeled a potential sarbecovirus recombination event, inserting the Khosta 2 RBD within the SARS-CoV-2 spike backbone, human serum from SARS-CoV-2 vaccinated individuals could no longer neutralize these pseudoviruses (60). The identification of human ACE2-compatible sarbecoviruses in Europe expands our appreciation for the geographic distribution of pandemic threats. A contemporary assessment of circulating viruses and their potential spillover risk will provide a strategic edge in pandemic preparedness.

## MERBECOVIRUSES

There are over 2,100 merbecovirus (previously known as betacoronavirus lineage C) entries listed on the NCBI virus database with over 300 non-MERS viral sequences representing substantial variation across the subgenus. To characterize cell entry for the merbecovirus subgenus, we developed the MerbecoType system using MERS-CoV as the spike backbone because of its well-established structural characterization and use in *in vitro* research (10, 77–79). However, unlike sarbecoviruses, the higher diversity among merbecoviruses provided a challenge for designing a modular MERS-based spike. Thankfully, a number of advances have allowed for this development: the RBD coordinates for MERS-CoV have been identified (77), and the spike structures for the bat merbecoviruses HKU4, HKU5, NeoCoV, and PDF-2180 were resolved (80–82). The discovery that NeoCoV and PDF-2180 used ACE2 as an entry receptor (80), unlike dipeptidyl peptidase 4 (DPP4) in the cases of MERS-CoV (77) and HKU4 (81), provided a target to confirm our ability to exchange RBDs and change receptor compatibility. A comparison of published spike sequences revealed two glycine residues, conserved in every merbecovirus spike sequence, that flank the RBD at positions 372 and 610. These residues are structurally located in the flexible loop region in the S1 domain at the base of the RBD (Fig. 1D). Replacing the MERS-CoV spike RBD with the region of PDF-2180 spike between these two glycines switched the MERS-CoV spike receptor use from DPP4 to ACE2 (61).

We next designed a panel of over 30 viral pseudotypes expressing chimeric spikes that represented the total diversity of merbecoviruses at the time. As previously seen with sarbecoviruses, analysis of the functional RBDs revealed indel patterns that could classify merbecoviruses into four major clades. However, these clades were less geographically constrained. Using our assay, clade 1 RBD viruses, which include MERS-CoV and HKU4, and clade 3 composed of NeoCoV and PDF-2180 were confirmed to use DPP4 and ACE2, respectively (61). HKU5 viruses, which were the vast majority of clade 2 viruses in our panel, were discovered to specifically use ACE2 from their host, *Pipistrellus abramus* bats (61). The ability to use ACE2 homologs by other clade 2 viruses has been confirmed in several recent studies (55, 83–86). Clade 4 viruses or “ErinCoVs” were discovered in *Erinaceus europaeus/amurensis* hedgehogs across Europe and China (87–97). In contrast to DPP4- and ACE2-dependent merbecoviruses, ErinCoVs utilized hedgehog aminopeptidase N (APN) for entry (62). Similar to the clade 2 RBD merbecoviruses like HKU5, the ErinCoV RBDs are remarkably species specific. Mutagenesis of the interface between ErinCoV RBD and APN from several species revealed multiple barriers that restrict the ErinCoVs ability to use APN from other hosts (62).

## EXPANDING THE CONCEPT

The RBD in betacoronavirus spike proteins is a single, contiguous domain capable of assuming its biologically relevant fold independent of the rest of the spike protein. This property has been highly advantageous for the study of coronavirus spike structure, as nearly all co-structures of spikes engaged with their receptor have been determined using solely the RBD. The structural independence of the spike RBD is also beneficial for the development of RBD-based vaccines and the RBD exchanging strategies employed in functional viromics approaches. Not all viruses engage with their host receptors through single domain interactions. For example, amino acids involved in receptor engagement for HIV-1 are distributed in multiple distinct regions in the Gp120 subunit (98). Similarly, filoviruses interact with their receptors through multiple loops throughout the N-terminal domain of the viral glycoprotein (99). Thus, a functional viromics approach to testing viral entry for some viruses would require identification of what regions can be exchanged, if individual receptor contact domains can be swapped, or if the entire region spanning all receptor contact domains should be exchanged. Current pseudotyping methods are limited to enveloped viruses. There are no widely adaptable approaches to test cell entry for different types of non-enveloped viruses, which employ the use of more complex, oligomeric capsid structural features to engage with their receptors.

The concept of functional viromics is not restricted to only measuring RBD interactions with receptors. We have shown that the chimeric spikes can also be used to measure antibody neutralization and vaccine resistance (60). While focusing only on the RBD limits experiments to only neutralizing responses, similar domain exchange strategies can be taken with other regions of spike.

Identification of new viral-host interactions will facilitate expansion of the approaches that can be taken experimentally. As mentioned earlier, the multiple steps of coronavirus cell entry are each mediated by separate domains within the spike gene. As researchers uncover host factors involved in these steps, viral spike function can be further screened across its other domains. For example, some coronaviruses have been shown to rely on sialic acids or heparin sulfate for cell attachment (44, 100, 101), while other viruses utilize proteinaceous host factors, such as glucose-regulated protein (GRP78) (102–108), dendritic cell-specific intercellular adhesion molecule-3-grabbing non-integrin (DC-SIGN), or liver/lymph node-specific intracellular adhesion molecule-3 grabbing non-integrin (L-SIGN). The approach we have taken for the RBD should be applicable to assessing viral entry, as it pertains to the use of these other attachment factors. Confounding this approach are the often-redundant roles some of these attachment factors may serve for multiple viruses as well as the challenges of modifying non-proteinaceous factors including sialic acids and other sugars. While transmembrane serine protease 2 (TMPRSS2) and cathepsin L are widely implicated in coronavirus spike processing, multiple proteases have been shown to facilitate entry as well (69, 72–74, 109–116). Thus, with multiple proteases performing redundant roles in spike processing, similar challenges arise with screening spike protease processing in a functional viromics approach.

Functional viromics is also not limited to only the virus side of virus-host interactions. Recent work has shown similar modular protein designs can be taken with host-cell factors, such as the sarbecovirus receptor, ACE2 (117). In this work, Roelle and colleagues demonstrated the region of ACE2 that engages with sarbecovirus RBDs can be exchanged with analogous regions of ACE2 orthologs from other species and utilized in viral entry assays. Liu and colleagues fused antibodies that were directed toward coronavirus spikes with cell-membrane-bound anchors to develop synthetic receptors. The resulting molecules facilitated viral entry and replication, albeit through interfaces with unclear biological relevance to natural virus infection (118). Nevertheless, synthetic receptor approaches could allow for in-depth intracellular characterization of viral replication.

Functional viromics may also be suitable for post-entry steps, although these viral-host interactions will require further definition and development of assays to screen the resulting phenotypes. For example, large-scale functional assays comparing different strains of human immunodeficiency virus (HIV-1) in their ability to counteract post-entry host restriction factors have been used to map protein interfaces and identify species barriers (119, 120). Mass spectrometry analysis of virally infected cells has demonstrated hundreds of possible virus-host interactions that occur during viral infection, which may prove invaluable to identifying the next targets for functional viromics screens (121, 122).

## IMPROVEMENTS TO PANDEMIC PREPAREDNESS

Although functional viromics approaches cannot predict the next pandemic, they can help prioritize viral discoveries based on key phenotypes involved in cross-species transmission. In the summer of 2021, new sarbecoviruses, called Khosta-1 and Khosta-2, were reported in *Rhinolophid* bats in Sochi National Park, Russia (123). Using our chimeric spike platforms, we found the Russian sarbecoviruses were capable of using human ACE2 for cell entry, and studies from other groups also identified this interaction through other means (52, 60, 117). Concurrently, in 2021, SARS-CoV-2 was reported to spill back into animals, with reports of the virus in domestic cats, other felid species in zoos (124–129), deer across North America (130–133), as well as mink farms in Europe (134–136). Because of these concerning reports of SARS-CoV-2 cross-species spillover, we generated a chimeric spike based on SARS-CoV-2 but with the RBD from Khosta-2. This chimeric spike resembling a possible recombinant form of a sarbecovirus resulting from SARS-CoV-2 spillover into Khosta-2-infected animals allowed us to assess cross-reactivity of human immune responses directed toward SARS-CoV-2. Using serum from patients who had received commonly available SARS-CoV-2 vaccines or who had recovered from infection with the latest circulating strains demonstrated little cross-reactivity or protection toward the chimeric spike, underscoring the need for broadly protective vaccines and therapeutics. In 2023, antiviral compounds that downregulate cell-surface ACE2 levels were shown to be broadly protective against all ACE2-dependent coronaviruses, including Khosta virus (137). In just 2 years, virus discovery rapidly progressed from sequence deposition to phenotype characterization, threat assessment, and antiviral drug development with small animal model validation. A broadened understanding of viral entry phenotypes and species receptor compatibility across the virome will also help in improving vaccine design. For example, our initial sarbecovirus entry screens were used to select antigens for mosaic nanoparticle vaccines that exhibited cross-protection against diverse sarbecoviruses both *in vitro* and *in vivo* (138). Altogether, functional viromics approaches, in tandem with surveillance and countermeasure development, represent a strong front against future pandemics.

Sarbecoviruses are only one possible source of pre-emergent viruses. Our MerbecoType system allowed us to demonstrate that while some HKU5 viruses are restricted in species’ ACE2 use, other HKU5 viruses remain a threat due to the ability to adapt further (61). Additionally, some newly identified HKU5 viruses have been reported to use mink ACE2 (84, 86). These findings have been further validated by the spillover events in farmed American mink, *Neogale vison*, in China (139). We have performed structural mutagenesis to identify molecular determinants in the RBD that permit some HKU5 viruses to use mink ACE2, as well as pinpoint a single amino acid change that is naturally circulating in HKU5 viruses and allows HKU5-like virus isolated from mink to use human ACE2 (140). These results showed that while HKU5 viruses may have been initially deemed less of a risk for zoonotic spillover from bats into humans, an intermediate host like mink could help mediate such a spillover. In further support of the risk HKU5 viruses may pose to humans, a second lineage of HKU5 viruses (HKU5-CoV-2) discovered in 2025 was shown to intrinsically possess human ACE2 compatibility (55, 140).

Structural mutagenesis of the interface between ErinCoV RBDs and European hedgehog APN has demonstrated multiple critical residues required for viral entry across other species (62). Importantly, our results show that currently published ErinCoVs have a limited ability to use APN homologs from other species, with human APN notably having multiple mutations that prevent interaction with these viruses (62). Thus, in contrast to HKU5 viruses, we believe our hedgehog coronavirus results demonstrate that ErinCoVs currently in circulation likely pose a limited risk to humans. As now observed for other betacoronaviruses, new discoveries may challenge this notion, necessitating additional viral surveillance.

## COMPUTATIONAL APPLICATIONS FOR FUNCTIONAL VIROMICS

**Fig 2:**
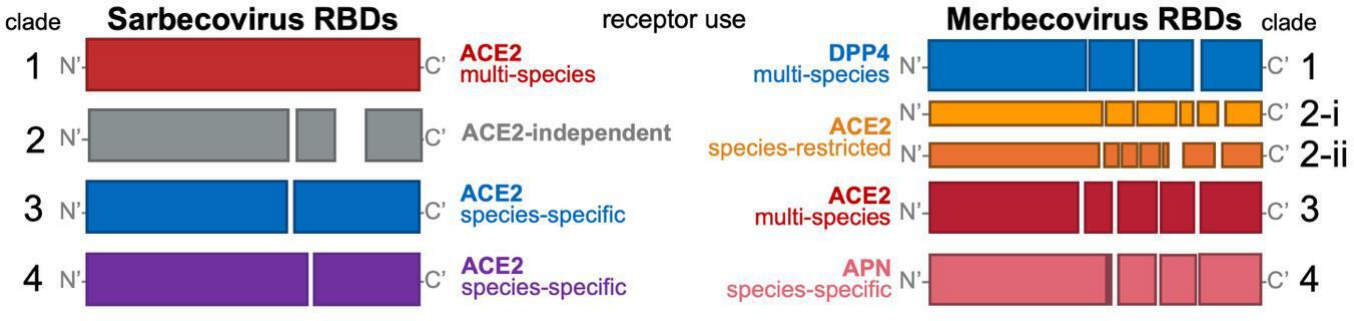
Phylogenetically and functionally defined coronavirus RBD clades. Schematic of RBD amino acid sequence indels with deletions visualized as gaps. Sarbecovirus and merbecovirus RBDs can be defined by characteristic indel patterns that underlie their phylogenetic assortment and resulting cell entry phenotypes determined by functional assays.

Even with functional viromics approaches, testing every newly discovered virus still requires physical lab efforts, personnel time, and financial support. Ideally, researchers could utilize our findings at the time of virus discovery to assess zoonotic risk. Screening dozens of uncharacterized viruses for cell entry phenotypes has shown that betacoronavirus RBDs share both phylogenetic and functional relationships. Using this information, we have defined various clades of RBDs for sarbecoviruses and merbecoviruses (Fig. 2). We have also shown sequence assessment of sarbecovirus RBDs can be used to deduce entry phenotype, even for viruses with unknown entry routes. Our analysis of ACE2-independent, “clade 2” sarbecoviruses resulted in the identification of sequence determinants for viruses that are both ACE2 independent and possess compatibility with human cells (57). Therefore, data sets on viral phenotypes as they relate to viral genotypes will be instrumental in developing and training advanced machine learning models of zoonotic risk. In the not-too-distant future, researchers will be able to access highly accurate predictions on cross-species spillover potential for newly discovered viral genomes, which will subsequently allow for rapid assessment of risk and, if needed, deployment or refinement of available countermeasures.
